# Deep learning segmentation and registration-driven lung parenchymal volume and movement CT analysis in prone positioning

**DOI:** 10.1371/journal.pone.0299366

**Published:** 2024-02-29

**Authors:** Hyungin Park, Soon Ho Yoon

**Affiliations:** Department of Radiology, Seoul National University Hospital, Seoul, Republic of Korea; Scuola Superiore Sant’Anna, ITALY

## Abstract

**Purpose:**

To conduct a volumetric and movement analysis of lung parenchyma in prone positioning using deep neural networks (DNNs).

**Method:**

We included patients with suspected interstitial lung abnormalities or disease who underwent full-inspiratory supine and prone chest CT at a single institution between June 2021 and March 2022. A thoracic radiologist visually assessed the fibrosis extent in the total lung (using units of 10%) on supine CT. After preprocessing the images into 192×192×192 resolution, a DNN automatically segmented the whole lung and pulmonary lobes in prone and supine CT images. Affine registration matched the patient’s center and location, and the DNN deformably registered prone and supine CT images to calculate the x-, y-, z-axis, and 3D pixel movements.

**Results:**

In total, 108 CT pairs had successful registration. Prone positioning significantly increased the left lower (90.2±69.5 mL, *P* = 0.000) and right lower lobar volumes (52.5±74.2 mL, *P* = 0.000). During deformable registration, the average maximum whole-lung pixel movements between the two positions were 1.5, 1.9, 1.6, and 2.8 cm in each axis and 3D plane. Compared to patients with <30% fibrosis, those with ≥30% fibrosis had smaller volume changes (*P*<0.001) and smaller pixel movements in all axes between the positions (*P* = 0.000–0.007). Forced vital capacity (FVC) correlated with the left lower lobar volume increase (Spearman correlation coefficient, 0.238) and the maximum whole-lung pixel movements in all axes (coefficients, 0.311 to 0.357).

**Conclusions:**

Prone positioning led to the preferential expansion of the lower lobes, correlated with FVC, and lung fibrosis limited lung expansion during prone positioning.

## Introduction

Prone positioning, defined as positioning patients upside down in a prone position, has gained interest as a non-invasive, cost-effective strategy for hypoxic intubated and non-intubated awake patients [1]. Prone positioning can reduce the pleural pressure gradient and increase aeration to dorsal lung segments, and since it was first applied by Bryan [2], several studies have succeeded in applying it to patients with respiratory distress, including patients infected with coronavirus disease 19. Indeed, prone ventilation improved gas exchange [3] and decreased mortality from acute respiratory distress syndrome (ARDS), especially when applied in the early phase of ARDS [4].

Clinical and experimental reports have consistently shown that prone positioning results in improved oxygenation [5]. Nevertheless, the pathophysiology remains uncertain, and a discordant perspective [5] suggests that the normally ventilated ventral lung regions would be better perfused in a prone position, improving ventilation-perfusion matching. Some have also suggested that the lung volume, especially in the usually dependent dorsal lung regions, would be redistributed in response to a position change, regardless of perfusion variations.

Furthermore, prone positioning has not been shown to benefit all patients with respiratory distress. In a study of patients with pulmonary fibrosis, the parameters of gas exchange or hemodynamics did not show significant improvement in response to prone positioning [6]. An experimental animal study also suggested that the effect of prone positioning might depend on the stage of lung injury and the duration of prior ventilation [7]. Few imaging studies have dealt with the physiological differences between the two positions [2, 6, 8] or experimentally compared CT lung attenuation [9]. Shin et al. evaluated the differences of quantitative CT-based image registration metrics of the lung, but the study only included 34 healthy individuals with sequential follow-up prone CT scans [10].

Therefore, this study aimed to analyze the volumetric and movement changes between the two positions with the help of deep neural networks (DNNs) in healthy controls and patients with varying degrees of pulmonary fibrosis.

## Materials and methods

This retrospective study was approved by the institutional review board of the institutional review board of Seoul National University Hospital (IRB No.: 2207-073-1339), and the requirement for informed consent was waived.

### Study population

We searched patients with the following inclusion criteria: (a) visited between June 2021 and March 2022, (b) suspected of having interstitial lung abnormalities or interstitial lung disease (ILD), (c) having full-inspiratory chest CT scans in supine and prone positions within 1year, (d) CT findings that remained unchanged between the supine and prone CT scans, and (e) with matching pulmonary function test (PFT) results after the prone CT. We excluded patients for whom supine and prone CT were registered incorrectly or lung segmentation by the neural network was imperfect.

A study coordinator collected the demographics and PFT results of the included patients from their electronic medical records.

### CT acquisition

All full-inspiratory prone chest CT scans were obtained using one of four different multi-channel CT scanners: Ingenuity (n = 96), IQon Spectral CT (n = 7), Brilliance iCT 256 (n = 3) and Brilliance 64 (n = 2)(Philips Healthcare, Best, The Netherlands). Most CT protocols were set at 120 kV, with the mAs calculated based on the set dose right index (DRI, 9 for supine acquisition and 6 for prone acquisition) and pitch of 0.6–1.5. The CT scans were scanned craniocaudally without contrast medium. CT axial images were reconstructed at 1-mm or 1.25-mm thickness (thin section, sharp kernel) and 3-mm thickness (thick section, standard kernel). Thin-section sharp kernel images were retrieved for further analysis.

### Visual CT analysis

One board-certified thoracic radiologist (5 years of clinical experience in chest CT interpretation) visually assessed the extent of fibrotic abnormalities under the supervision of a senior thoracic radiologist (17 years of clinical experience) in a lung window setting (window level, -600; window width, 1500) on a picture archiving and communication system (INFINITT Healthcare, Seoul, South Korea). The fibrotic abnormalities comprised ground-glass opacity (GGO), consolidation, reticulation, and honeycombing on supine CT images. The radiologist was blinded to the patient’s clinical information and the results of the PFT. The fibrotic abnormalities were evaluated as the sum of GGO, consolidation, reticulation, and honeycombing in units of 10% of the whole lung. Patients were dichotomized as having < 30% or ≥30% fibrosis to render an extensive fibrosis group [11].

The radiologist also assessed high-resolution chest CT patterns, according to the recently updated 2022 American Thoracic Society (ATS)/European Respiratory Society (ERS)/Japanese Respiratory Society (JRS)/Latin American Thoracic Association (ALAT) guideline for idiopathic pulmonary fibrosis [12].

### Lung segmentation, image registration, and quantification

The Advanced Normalization Tools in Python package (ANTsPy, v0.2.2; Python Software Foundation) was used for semi-automatic image preprocessing, including image resizing and affine registration. Then, a commercially available segmentation software (MEDIP PRO v2.0.0.0; MEDICALIP, Seoul, Korea) was used for segmentation of the whole-lung masks (i.e., right upper, right middle, right lower, left upper and left lower lobes), and a publicly available deformable image registration DNN (LungReg) was used for deformable registration.

Supine and prone CT images were resized to 192×192×192 resolution using ANTsPy, and CT attenuation was scaled by a factor of 1/3000. Whole-lung and lobar masks in prone and supine CT images were segmented by the previously trained U-Net on MEDIP PRO [13]. The senior radiologist, blinded to clinical information, reviewed and determined whether the segmentation masks perfectly matched the bilateral lungs and pulmonary lobes on the resized CT images.

First, whole-lung masks in prone CT images were affine-registered (translation, rotation, scaling, and no shearing) to whole-lung masks in the corresponding supine CT images. This step, which aligned the centers of the supine and prone CT images, allowed us to solely assess positional lung changes in the following deformable registration while excluding differences originating from different centering. Then, to predict a spatial displacement map, supine and affine-registered prone CT images were propagated to the priorly trained DNN, LungReg. Finally, via a spatial transform layer, we extracted a spatial displacement map in the deformable registration between the affine-registered prone CT images and the supine CT images.

From the spatial displacement map, whole-lung movements were measured by the three-dimensional (3D) Euclidean distance in millimeters between supine and affine-registered prone CT images. Hue, saturation, and value color and quiver maps were also used to visualize whole-lung movement. Hue, saturation, and value were used to represent the movement of left-right (x), anterior-posterior (y), and superior-inferior (z) directions, respectively.

### Statistical analysis

The paired t-test was performed to analyze lung and lobar volume differences between the supine and prone positions. The independent t-test was used to compare volume changes in each position between patients with mild fibrosis (<30%) and patients with severe fibrosis (≥30%).

The Spearman correlation coefficient was used to examine the correlation between the visual assessment of fibrosis extent and the volume changes. We also examined the correlations between forced vital capacity (FVC) and lung lobar volume changes and between FVC and pixel movements.

All statistical analyses were performed using SPSS version 27.0 (IBM Corp., Armonk, NY, USA). A two-sided *P* value < 0.05 was considered to indicate a statistically significant difference.

## Results

### Patient demographics

**Fig 1:**
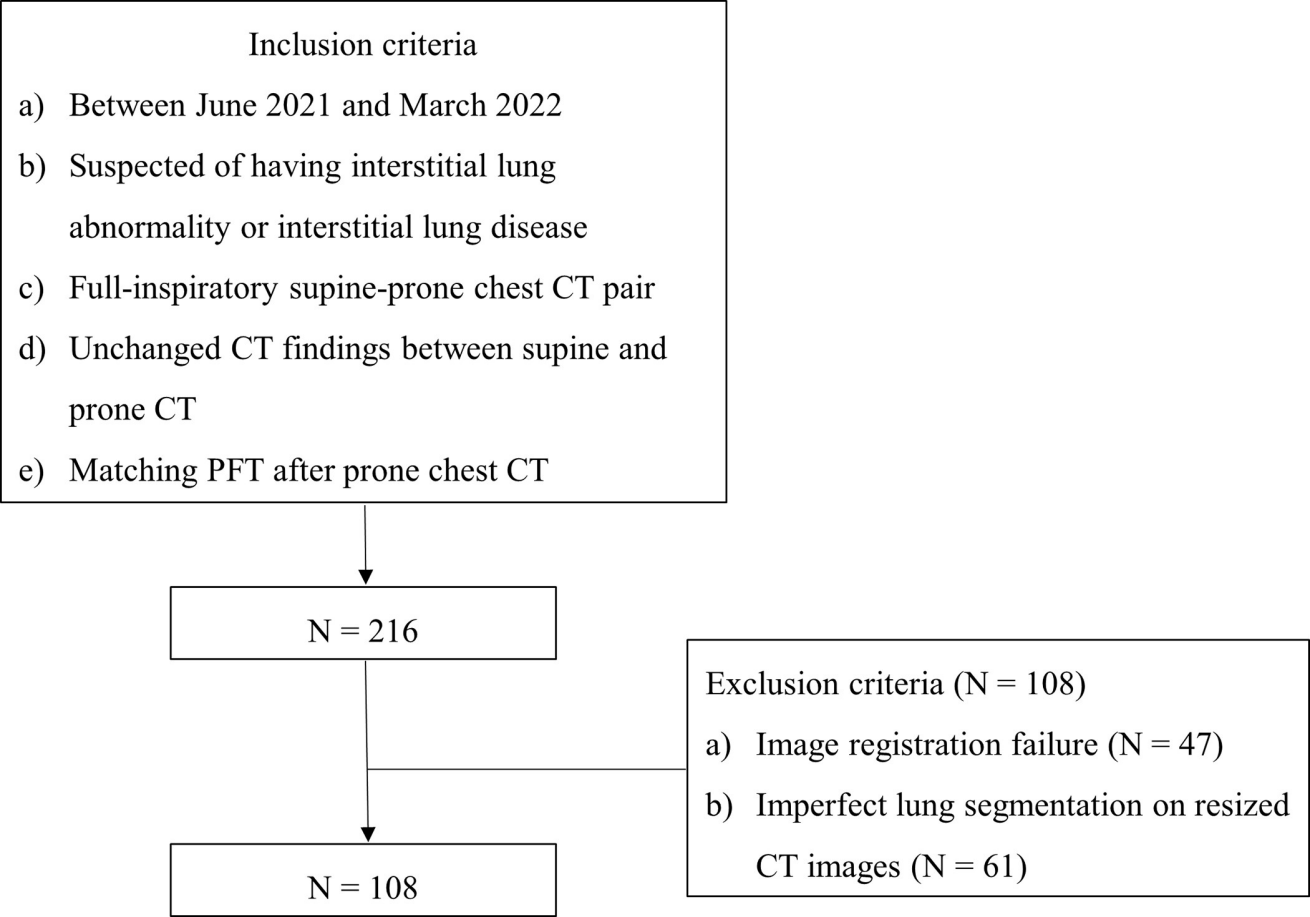
Flow chart of patient enrollment process. PFT: pulmonary function test.

Among the screened 216 patients, 108 were excluded due to failure of image registration (n = 47), and inappropriate lung segmentation from the resized CT images (n = 61). Finally, a total of 108 patients (mean age, 68.2 ± 12.4 years; male:female, 64:44) were included in the study. The patient demographics and enrollment process are summarized in Table 1 and Fig 1, respectively.

**Table 1:** Patient demographics.

		Total (n = 108)	Normal (n = 12)	UIP or probable UIP (n = 35)	Indeterminate or Alternative for UIP (n = 61)	*P*-value
**Age (year)** *		68.2±12.4	64.7±16.3	66.3±13.2	70.0±10.9	
**Sex** ^†^	Male	64 (59%)	7 (58%)	23 (66%)	34 (56%)	
	Female	44(41%)	5(42%)	12(34%)	27(44%)	
**Smoking history** ^†^	Never	50(46%)	6(12%)	13(26%)	31(62%)	
	Former	48(44%)	6(13%)	19(40%)	23(48%)	
	Current	10(9%)	0(0%)	3(30%)	7(70%)	
**Pack-years** *		14.0±20.3	13.7±18.3	14.2±15.2	16.9±23.3	0.230
**PFT** *	FVC (L)	2.8±0.8	3.0±1.0	2.8±0.8	2.7±0.8	0.424
	FVCpred (L)	85.3±18.5	89.8±20.8	81.5±17.8	86.5±18.4	0.299
	FV1pred (L)	93.5±22.6	87.7±32.2	82.2±21.0	95.4±21.5	0.515
	FEV1/FVC (%)	76.2±9.8	66.7±13.4	78.9±6.9	76.5±9.4	0.001
CT extent (%)*	Total	33.4±20.1	0	41.7±15.8	35.3±17.7	0.000
	GGO	15±11.2	0	14.3±8.5	18.4±11.1	0.000
	Consolidation	3.5±4.8	0	4.3±5.0	3.8±4.9	0.022
	Reticulation	12.9±9.2	0	17.4±7.4	12.8±8.6	0.000
	Honeycombing	2.0±5.1	0	5.7±7.4	0.3±1.8	0.000

Note.–UIP = usual interstitial pneumonia, PFT = pulmonary function test, FVC = forced vital capacity, FEV1 = forced expiratory volume in one second, pred = percent predicted, GGO = ground-glass opacity. Data in parentheses indicate the proportion of patients.

*Data indicate mean ± standard deviation values

^†^Data indicate number of individuals (proportion among entire individuals) values

The study population consisted of patients with usual interstitial pneumonia (UIP) pattern (n = 16), probable UIP pattern (n = 19), indeterminate for UIP pattern (n = 19), alternative diagnosis (n = 42), and normal (n = 12) according to the Fleischner Society guidelines. Almost all of the enrolled patients (n = 102) underwent prone and supine CT scans on the same day, whereas 6 patients had prone and supine CT scans taken on different days, with a median interval of 343 days (interquartile range, 199–360). The median interval between PFT and the prone CT scan was 0 days (interquartile range, 0–7).

### Visual assessment

The results of the visual assessment are summarized in Table 2. The extent of GGO was visually assessed as 0% (n = 18), 10% (n = 45), 20% (n = 25), 30% (n = 14), 40% (n = 5), and 50% (n = 1). The consolidation extent was assessed as 0% (n = 70), 10% (n = 38), and more than 20% (n = 0). Reticular opacity was assessed as 0% (n = 20), 10% (n = 49), 20% (n = 29), 30% (n = 8), 40% (n = 2), and more than 50% (n = 0). Finally, the extent of honeycombing was visually assessed as 0% (n = 91), 10% (n = 12), 20% (n = 5), and more than 30% (n = 0). Overall, 36% of the patients (n = 39) were categorized as having mild fibrosis, and 64% of the patients (n = 69) were categorized as having severe fibrosis.

**Table 2:** Visual CT assessment of fibrotic abnormalities.

	Total	Ground-glass opacity	Consolidation	Reticular opacity	Honeycombing
**0%**	12 (11%)	18 (17%)	70 (65%)	20 (19%)	91 (84%)
**10%**	7 (6%)	45 (42%)	38 (35%)	49 (45%)	12 (11%)
**20%**	20 (19%)	25 (23%)	0	29 (27%)	5 (5%)
**30%**	21 (19%)	14 (13%)	0	8 (7%)	0
**40%**	12 (11%)	5 (5%)	0	2 (2%)	0
**50%**	19 (18%)	1 (1%)	0	0	0
**60%**	13 (12%)	0	0	0	0
**70%**	3 (3%)	0	0	0	0
**80%**	0	0	0	0	0
**90%**	1 (1%)	0	0	0	0

Note. Data indicate number of individuals (proportion among entire individuals) values.

### Quantitative assessment

The Dice similarity coefficients between prone and registered supine lung masks were 0.97±0.02 for the total lung, 0.97±0.02 for the right lung, and 0.97±0.03 for the left lung. Right lower lobe and left lower lobe volume significantly increased in the prone position (mean ± SD: 52.5 ± 74.2 mL; 95% CI, 38.6–66.4 mL; *P* = 0.000 and 90.2 ± 69.5 mL; 95% CI, 77.2–103.2 mL; *P* = 0.000, each). On the contrary, the right middle lobe volume significantly decreased in the prone position (mean ± SD: -33.9 ± 44.8 mL; 95% CI, -42.3 to -25.5 mL; *P* = 0.000). The whole lung volume, right and left lungs, and both upper lobes did not show statistically significant differences between the two positions (Table 3).

**Table 3:** Lung volume change in prone positioning from supine positioning.

	Mean	SD	95% CI	*P*-value
**Whole lung (mL)**	85.4	235.8	41.3 to 129.6	.000
**Right lung (mL)**	3.2	134.8	-22.1 to 28.4	.803
**Left lung (mL)**	82.2	110.5	61.5 to 102.9	.000
**RUL (mL)**	-15.5	54.4	-25.7 to -5.3	.003
**RML (mL)**	-33.9	44.8	-42.3 to -25.5	.000
**RLL (mL)**	52.5	74.2	38.6 to 66.4	.000
**LUL (mL)**	-8.0	61.9	-19.5 to 3.6	.177
**LLL (mL)**	90.2	69.5	77.2 to 103.2	.000

Note.–RUL: right upper lobe, RML: right middle lobe, RLL: right lower lobe, LUL: left upper lobe, LLL: left lower lobe, SD: standard deviation, CI: confidence interval

### Vector analysis

The average maximum whole-lung pixel movements between prone and supine positions in the x-, y-, and z- axes and 3D plane were 1.5 cm, 1.9 cm, 1.6 cm, and 2.8 cm, respectively. On the 3D plane, the average maximum whole-lung pixel movements showed significant differences among patients without fibrosis (3.9±1.1 cm), mild fibrosis (3.2±1.2 cm), and severe fibrosis (2.5±0.9 cm) (*P*<0.001). The difference persisted even when patients were dichotomized into those with none to mild lung fibrosis and those with severe lung fibrosis (3.4±1.2 cm, 2.5±0.9 cm, *P* = 0.000). When categorized into normal, UIP or probable UIP patterns, and indeterminate for UIP or alternative diagnosis patterns according to the ATS/ERS/JRS/ALAT guideline, the difference in the 3D pixel movements of the whole lung was also significant (3.9±1.1 cm, 2.6±0.8 cm, and 2.8±1.2 cm, respectively; *P* = 0.001). The results are summarized in Table 4.

**Table 4:** Comparison of whole-lung pixel movements between prone and supine positions.

	Total (n = 108)	No fibrosis (n = 12)	Mild fibrosis (n = 27)	Severe fibrosis (n = 69)	*P*-value	None to mild fibrosis (n = 39)	Severe fibrosis (n = 69)	*P*-value	No fibrosis (n = 12)	UIP or probable UIP (n = 35)	Indeterminate or alternative for UIP (n = 61)	*P*-value
**3D plane (cm)**	2.9±1.1	3.9±1.1	3.2±1.2	2.5±0.9	0.000	3.4±1.2	2.5±0.9	0.000	3.9±1.1	2.6±0.8	2.8±1.2	0.001
**X-axis (cm)**	1.5±0.4	1.8±0.5	1.6±0.3	1.4±1.0	0.001	1.7±0.4	1.4±1.0	0.001	1.8±0.5	1.5±0.5	1.4±0.4	0.016
**Y-axis (cm)**	1.9±0.6	2.3±0.6	2.1±0.7	1.7±0.5	0.000	2.2±0.7	1.7±0.5	0.000	2.3±0.6	1.7±0.6	1.9±0.6	0.011
**Z-axis (cm)**	1.6±0.6	2.1±0.7	1.7±0.7	1.5±0.5	0.004	1.8±0.7	1.5±0.5	0.014	2.1±0.7	1.5±0.5	1.6±0.6	0.009

Note.–Data indicate mean ± standard deviation values.

UIP: usual interstitial pneumonia, 3D: three-dimensional

### Volume change in pulmonary fibrosis

**Fig 2:**
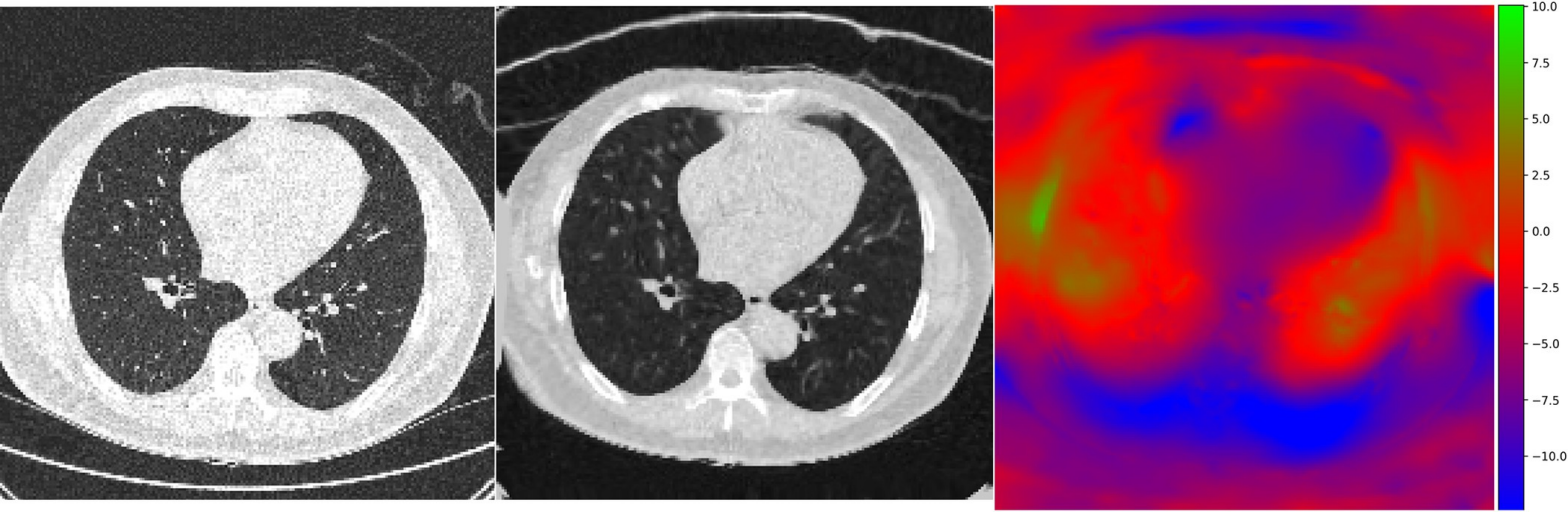
Representative pixel movement in deformable registration between supine and prone CT scans in a 71-year-old man without pulmonary fibrosis. Using ANTsPy, the CT images were resized into 192×192×192 resolution. After affine registration and deformable registration of both supine (A) and prone (B) images, the spatial displacement map was calculated. Whole-lung movement between the supine and prone positions was measured by the 3D Euclidean distance in millimeters. On a HSV map (C), there are variable movements in bilateral lungs, as color coded ranging from green to red. Note.–ANTsPy: Advanced Normalization Tools in Python package, 3D: three-dimensional, HSV: hue-saturation-value.

**Fig 3:**
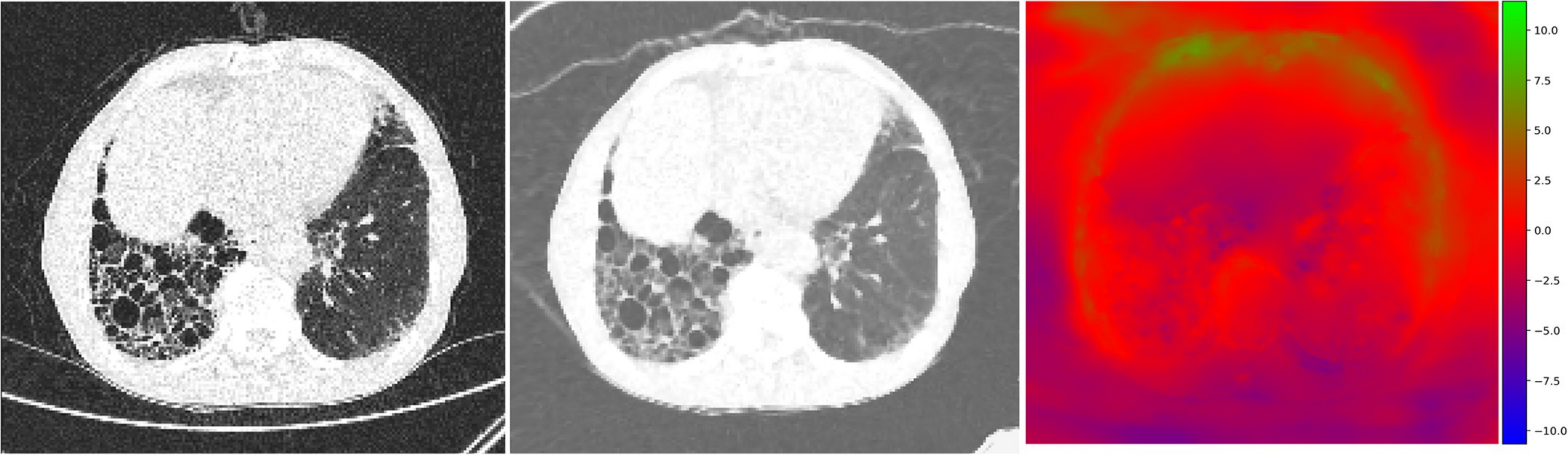
Representative pixel movement in deformable registration between supine and prone CT scans in a 76-year-old man with asymmetric pulmonary fibrosis. The 192×192×192 resized supine (A) and prone (B) images show a UIP high-resolution CT pattern with right lower lobe–dominant asymmetric subpleural reticulation, GGO, and honeycombing. On a HSV map (C), there are few movements in bilateral lungs, as color coded predominantly in red. Note.—UIP: usual interstitial pneumonia, GGO: ground-glass opacity, HSV: hue-saturation-value.

Patients with severe fibrosis (visual fibrosis degree ≥ 30%) showed smaller volume changes than those with mild fibrosis (*P*<0.001). The severe fibrosis group also showed smaller maximum pixel movements in all axes (*P* = 0.000 to 0.007) (Figs 2 and 3).

The Spearman correlation coefficient between the left lower lobe volume change and the visual assessment of fibrosis of the whole lung was -0.257 (*P* = 0.006).

### Correlation with PFT

**Fig 4:**
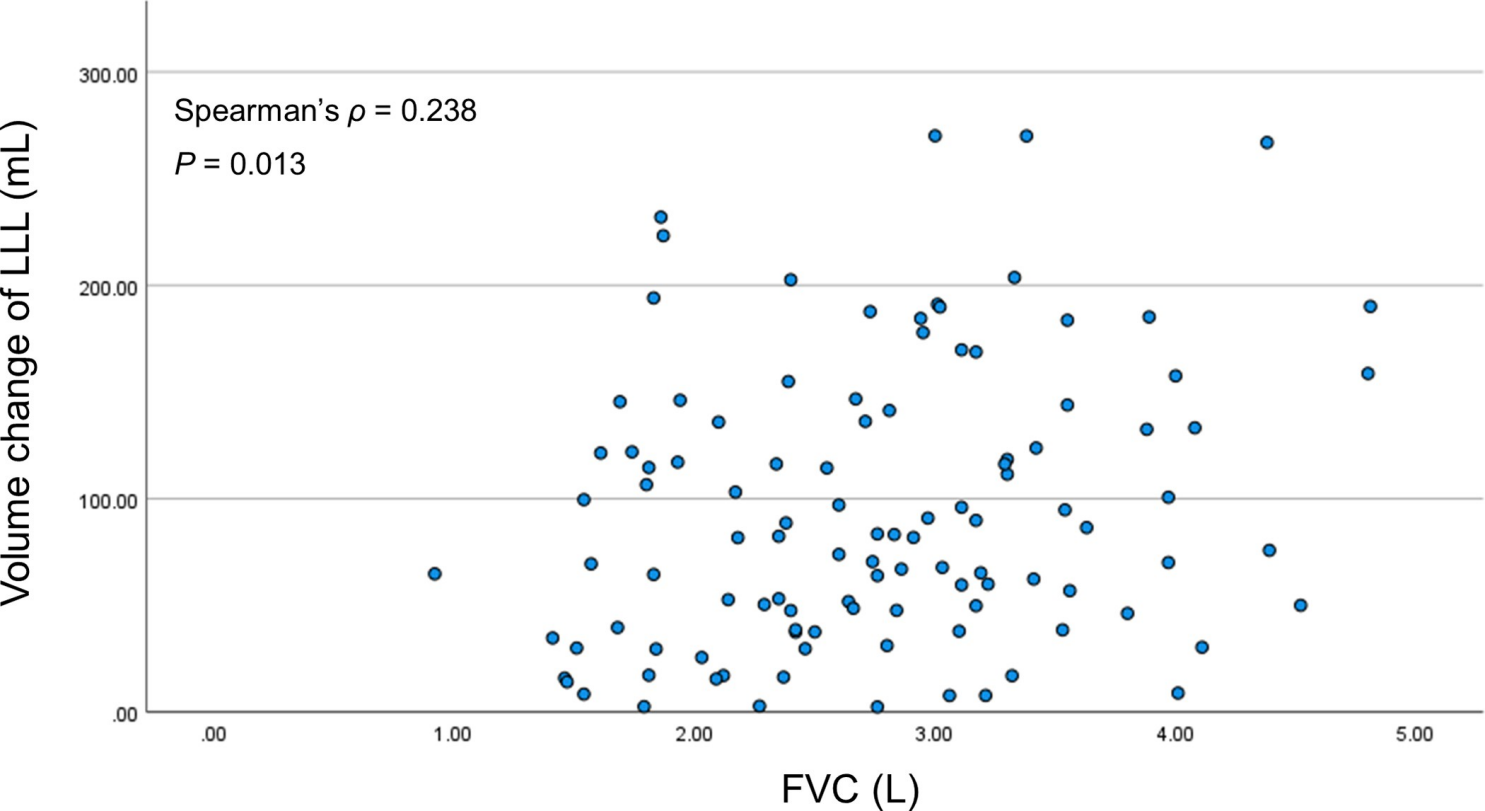
Correlation between FVC and the volume change of the left lower lobe. FVC: Forced vital capacity, LLL: left lower lobe.

The left lower lobe showed a positive correlation between FVC and the lung volume change, with a Spearman correlation coefficient of 0.238 (*P* = 0.013) (Fig 4).

**Fig 5:**
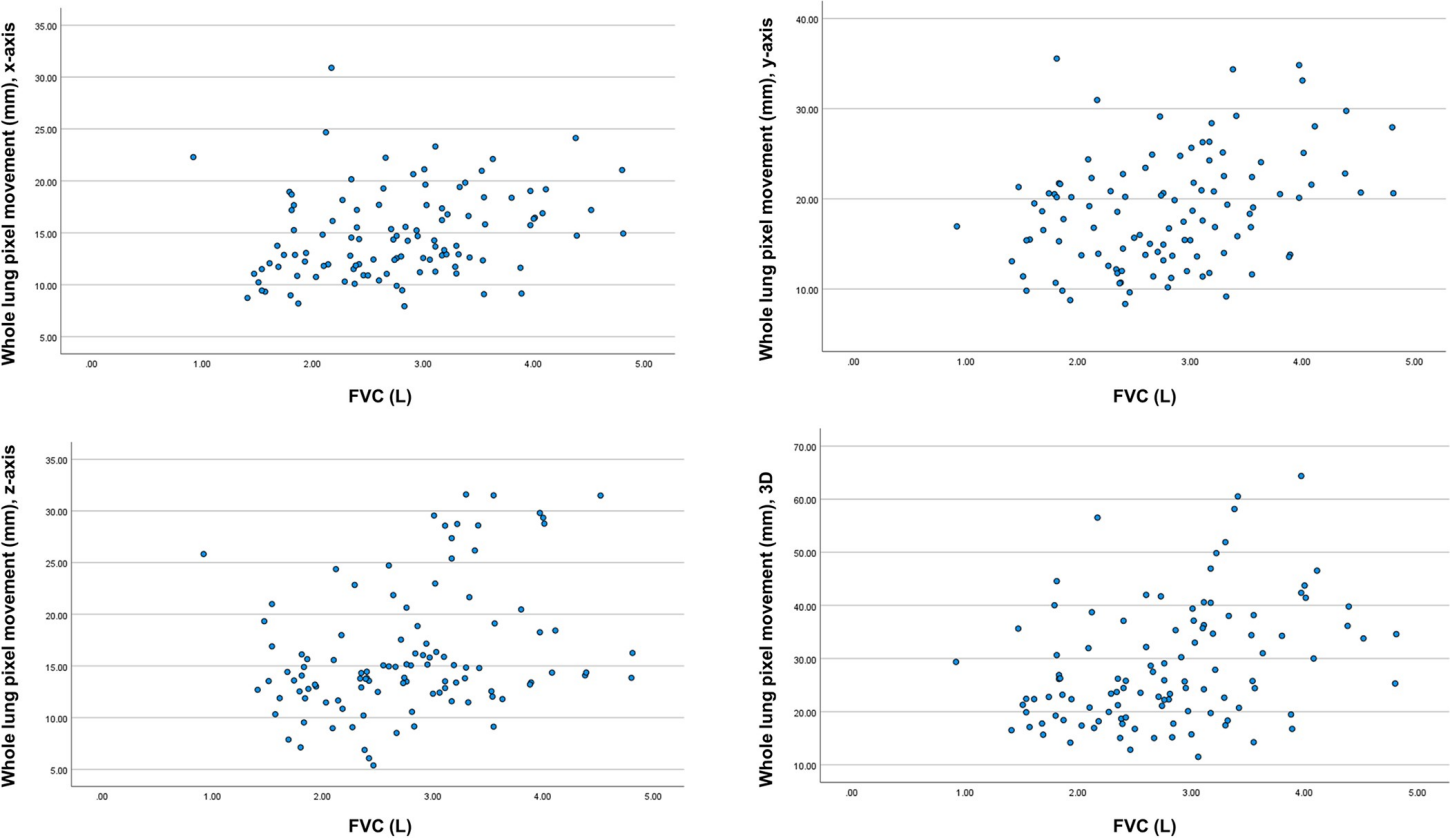
Correlation between FVC and whole lung pixel movement in the (A) x-, (B) y-, and (C) z-axes and (D) 3D plane. 3D: 3-dimensional, FVC: forced vital capacity.

There was a positive correlation between FVC and the maximum whole-lung pixel movements in all three axes and the 3D plane, with coefficients of 0.311, 0.315, 0.320 in the x-, y-, and z-axis, respectively (all *P*<0.05) and 0.357 on the 3D plane (*P* = 0.000) (Fig 5).

## Discussion

In this study, prone position increased both lower lobe volumes and decreased the right middle lobe volume significantly compared with the supine position. These volumetric changes are consistent with a previous study finding that lung inflation and recruitment during the position change are primarily localized in the dorso-caudal lung by investigating CT attenuation differences between the supine and prone positions [14]. Those researchers also concluded that the presence of opacifications in the dorsal lung is a key indicator of the therapeutic and protective effects of prone positioning [14].

Patients with severe lung parenchymal fibrosis showed smaller volume changes and pixel movements between supine and prone positions than patients with mild fibrosis. UIP/probable UIP patients with lung fibrosis in this study had subpleural and basal fibrosis corresponding to the dorsal lung [12]. Furthermore, in the subgroup analysis, the lung pixel movements of the whole lung on the x-, y-, and z-axes and the 3D plane were significantly lower in patients with UIP and probable UIP patterns. Our findings support that dorsal lung recruitment is vital in prone positioning, and idiopathic pulmonary fibrosis (IPF) patients with predominant dorsal fibrosis can recruit preserved lung areas by prone positioning to a lesser extent, leading to limited respiratory benefits from prone positioning.

Previous studies have mainly focused on the timing of the initiation of prone positioning, suggesting that prone positioning in ARDS patients needs to be applied early in the disease course [2]. The famous clinical trial (PROSEVA) concluded that prone positioning should be applied to ARDS patients within 72 hours in the disease course to maximize the clinical benefit [4]. The complex injury mechanism to the lung during the process of ARDS is not fully explained by fibrotic changes alone, but the physiologic difference between early ARDS and late ARDS is likely to be similar to that between non-fibrotic and fibrotic ILD patients, with decreased lung compliance and less recruitability. This might explain the result of smaller volume recruitment and pixel movement in patients with severe fibrosis in our study.

We also found that patients with better pulmonary function, represented as higher FVC values, had larger lung volume changes, especially in the left lower lobe. A similar correlation was also noted in the movement analysis, between higher FVC values and greater whole-lung pixel movement. PFT, which is routinely obtained in clinical practice, is considered to be a marker for pulmonary disease severity and a predictor of mortality. In particular, the percent predicted FVC has historically predicted survival in large cohort studies [15, 16]. Arcadu et al. reported that FVC was inversely correlated with a global assessment of interstitial abnormality [17]. This might explain our results, as lower FVC values correspond to severe lung parenchymal fibrosis, which was correlated with smaller volume change.

Our study had limitations. First, this study was retrospective, with a relatively small number of patients at a single institution, warranting further validation. Second, a considerable proportion of patients were excluded due to registration failure. In addition, the DNN provided imperfect lung segmentation, as the resized CT in-plane dimension (192×192) was different from the trained CT in-plane dimension (512×512). However, the registration process between supine and prone CT images is in its early steps with many challenging obstacles. Our results showed that this sophisticated task was feasible and could be improved further in the future. Third, the pixel attenuation change in the prone position could not be evaluated due to the lower radiation dose in the prone CT scan protocol (DRI 6, compared to DRI 9 for supine CT scans). Considering the well-known carcinogenic effects of ionizing radiation, avoiding unnecessary radiation exposure to patients would be inevitable in real clinical practice [18, 19]. Fourth, most patients underwent a prone CT scan just after obtaining a supine CT scan on the same day. Our prone positioning procedure might have been too short to fully reflect the effect of prolonged prone positioning in practice, since it is recommended to perform prone positioning for at least 16 hours per day [2]. Our result warrants validation to examine whether volume changes of dependent lungs in a short interval sufficiently reflect the therapeutic benefit of prone positing in practice.

## Conclusions

In conclusion, prone positioning led to an expansion of the lower lobes, which was correlated with FVC, and lung fibrosis limited lung expansion during prone positioning. Lower lobe recruitment in prone positioning is quantifiable on CT images and can be a new imaging biomarker for assessing lung function in ILD.
